# Ciltius, Altius, Fortius! Our Olympic games: simulation training for potential casualties massive influx during Paris 2024!

**DOI:** 10.1186/s44158-024-00220-z

**Published:** 2025-01-03

**Authors:** Myriam Lamamri, Raphaëlle David, Emmanuel Weiss, Mathilde Holleville

**Affiliations:** 1 Département d’anesthésie Réanimationéanimation, DMU PARABOL, AP-HP, Hôpital Beaujon, Clichy, France; 2 Université Paris-Cité, Inserm, Centre de Recherche Sur L’inflammation, UMR 1149, Paris, France; 3 Department of Anesthesiology and Critical Care, Beaujon University Hospital, 100 Boulevard du General Leclerc, Clichy, 92110 France

To the Editor,

## Ciltius: Being Ready!

Since the Paris attacks in 2015, exceptional health situation management has no longer been hypothetical on French territory, but unfortunately a reality in many countries [1]. Our anesthesia and intensive care department, based in a level 1 trauma center hospital in the Paris region, consists of 44 physicians. Despite our expertise in treating severe trauma patients (600 per year), we have limited experience in handling an injured patients’ massive influx.

**Fig. 1:**
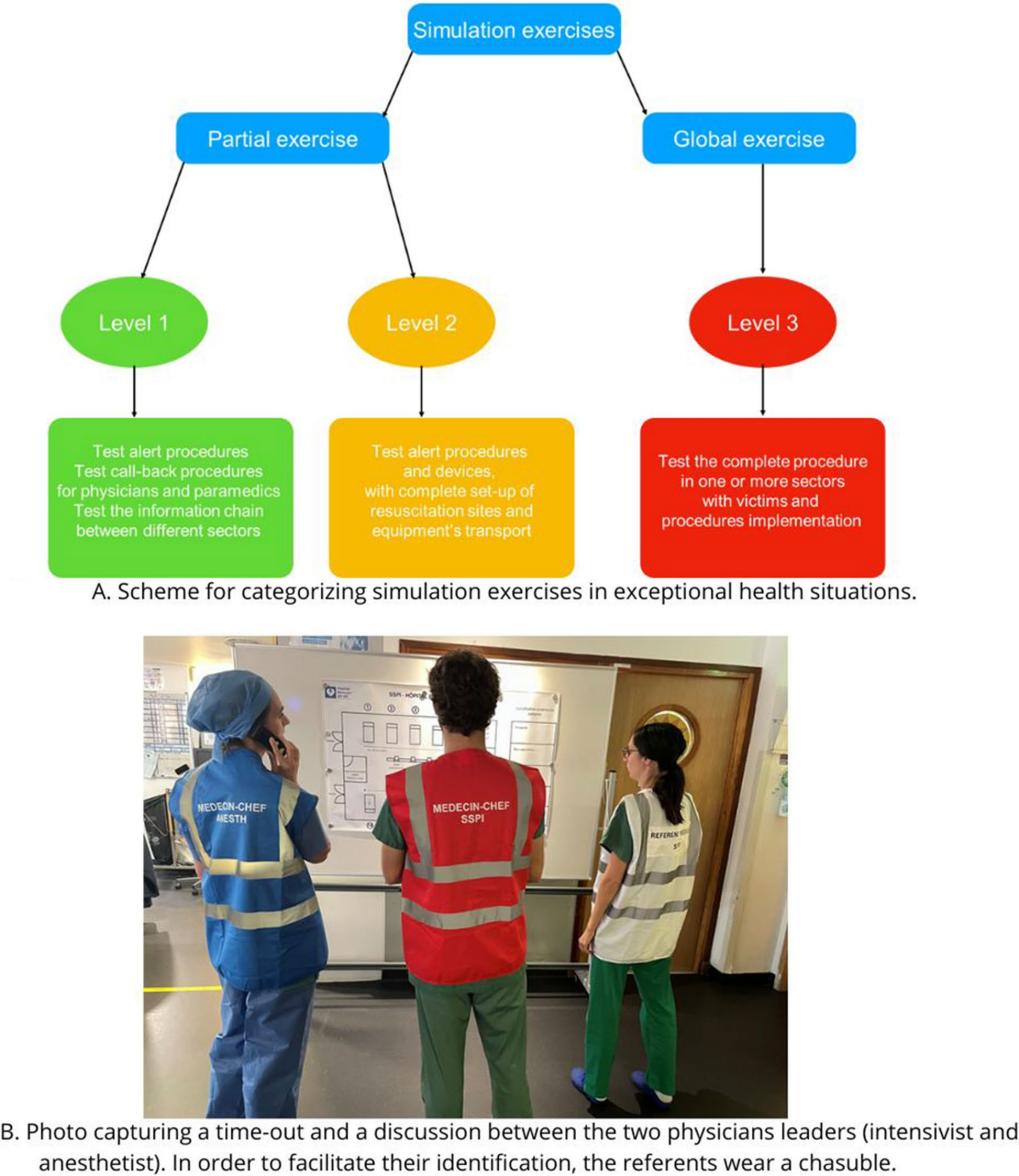
Simulation exercise categories and team leadership. **A** illustrates the categorization and objectives of our simulation exercises. **B** depicts a team timeout, showcasing a discussion between medical leaders (anesthesiologist and intensivist) and a nurse leader, easily identifiable by their distinctive vest

Anticipating the 2024 Paris Olympic Games, we intensified our preparations starting in 2022. Driven by our academic society and the French Ministry of Health, we updated our procedures and formalized a simulation training plan, including various levels of exercises based on simulation categorization (Fig. 1A) [2]. Our approach focused on monthly response simulations to test specific operating circuits and larger-scale exercises to evaluate the entire crisis management system.

## Altius: Climbing the steps

To ensure that all staff are prepared for exceptional healthcare situations, we conducted exercises both day and night, involving multidisciplinary teams from emergency, anesthesia-intensive care, and surgery departments.

During level 1 exercises, we evaluated our alert procedures. To assess real-time availability and mobilization time, we created a dedicated group using a non-institutional instant messaging platform. This platform is less vulnerable to cyberattacks and offers practical advantages. We conducted surveys to determine how quickly physicians could return to the hospital after an alert.

Daytime alert tests yielded high response rates (> 80%), allowing us to assemble medical reinforcements within 30 min. However, nighttime tests revealed a low response rate (3%) due to silent smartphone modes. Given the decline in landline use, we anticipate the need for direct phone calls during night-time emergencies to reach staff. Unfortunately, existing hospital alert systems cannot override silent mode, highlighting a need for alternative solutions. The issue is still pending. Despite living in a hyper-connected society, many people still rely on silent mode to disconnect from work.

In an effort to rectify this situation, we have engaged in discussions with hospital management. Two proposed remedies include a comprehensive staff education initiative emphasizing the critical nature of being contactable during the Olympic Games, and the implementation of a protocol requiring the crisis unit to conduct multiple attempts to reach staff members in order to disable silent mode.

During level 2 exercises, we focused on refining procedures from the alert stage to operating theater organization. These exercises highlighted the need for improved resuscitation site organization. To address this, we implemented a checklist for each site to streamline preparation and ensure equipment availability. This checklist significantly reduced the setup time for resuscitation sites prior to casualties arrival. Specifically, within 15 min of an alert, all equipment was in place at 8 resuscitation sites. Following a debrief with the nursing teams overseeing the checklist to enhance efficiency, we introduced dedicated cabinets for the organized storage of all required materials.

We established operational teams composed of nurses and physicians, easily identifiable by their matching vests. Our medical team comprises forty-four physicians with dual training in anesthesia and intensive care. While we initially considered a model where each physician would follow a patient throughout their care journey, we ultimately opted for a division into two distinct teams based on their primary daily responsibilities: intensive care or intra-operative management. This approach was deemed more efficient and supportive for physicians working in high-stress environments. Indeed, post-exercise evaluation revealed that unfamiliarity with workspaces led to increased cognitive load among professionals, potentially compromising the efficiency of processes, especially in emergency patient care. Therefore, a physician-nurse pairing system was implemented for both resuscitation and surgical procedures. Formal handovers, including written and verbal components, were instituted to facilitate patient transfers between these settings.

Level 2 exercises facilitated the identification of areas for improvement in interdepartmental coordination. We implemented and validated a new procedure with the blood bank to ensure safe and expedited blood product transfusion, including a non-nominative delivery system for packed red blood cells and fresh frozen plasma. A blood product delivery consisting of four units each of O + and O − packed red blood cells, as well as four units of fresh frozen plasma. Upon activation of the alert, the blood products, stored in a temperature-monitored bag, are transported to the trauma resuscitation area. Patients in need of transfusion can promptly receive the necessary components. To ensure the safe and timely delivery of blood products, the procedure was tested with a dedicated transport team. This team was specifically trained in handling blood products and equipped with temperature-controlled containers.

In mass casualty incidents, the high demand for medications can lead to a rapid depletion of the on-site pharmacy’s supply. Our exercises demonstrated a marked decrease in our intravenous fluid reserves. In collaboration with the hospital pharmacy, we developed a list of essential drugs, prioritizing sedatives, vasopressors, and intravenous fluids. A “mobile pharmacy box” was designed for rapid delivery within 30 min of an alert. These two technical aspects were crucial to implement as they form the foundation of severe trauma patient care. Their application during exercices has improved the transportation and packaging of blood products and drugs, ensuring the most rapid and secure delivery possible.

Level 2 exercises also allowed us to assess the feasibility of reorganizing surgical activities during mass casualty events. Our protocol mandates the rapid curtailment of ongoing surgeries and a swift assessment of available operating rooms. Although we did not simulate actual surgical procedure shortening, we identified potential delays in freeing up operating theaters, particularly during daytime hours. To discuss, this challenge, we organized and convened surgeons to a seminar on damage control surgery and surgical triage.

Over the past year, we have accomplished three level 3 exercises: during the afternoon, evening, and one in the middle of the night. Carrying out an exercise on this scale is no easy task. Human and material resource mobilization generates considerable costs and constraints. However, benefits in terms of learning, practice evaluation, and team building are inestimable [3]. A large-scale night exercise was conducted in collaboration with police headquarters, the Emergency Medical Assistance Service, the fire brigade, the regional health agency, and multiple departments within our hospital. The scenario involved a grandstand collapse during a sporting event, resulting in 200 casualties, including 10 deaths, 50 relative emergencies, 40 absolute emergencies, and 100 individuals involved in pre-hospital care. The key findings and lessons learned were:

### Interagency collaboration

The exercise demonstrated strong collaboration between social and medical services, both within and outside the hospital. Communication being the most important element in this kind of event, we created a link between teams by adopting two medical leaders who should work hand in hand. The intensivist leader oversees victims’ massive influx reception and resuscitation management. The anesthesiologist leader coordinates patient flow in the operating theaters. These two referents must not take care of patients. They were solely responsible for organizing patient flows within our department (with a chief intensive care nurse and a chief anesthetic nurse), and were in charge of communication with the rest of the hospital's departments (Fig. 1B). They also played a coordinating role with the crisis unit, both in transmitting information (medical needs, missing human resources) and in adapting the treatment chain to the evolution of the disaster.

### Procedural evaluation

We assessed our ability to execute the entire procedure sequence, including crisis unit activation, staff recall, bed availability, and patient influx management. Repeated level 2 exercises enabled all teams to familiarize themselves with the procedures and apply them during level 3 exercises. A key learning was the importance of repetitive practice of procedural chains to achieve mastery.

### Identity traceability

Surprisingly, patient identity traceability proved to be a more significant challenge than medical care. Errors in identification can have detrimental consequences for both casualties and their families.

### Dedicated personnel

To address this issue, we assigned a paramedic to the “admissions office” to ensure accurate patient identification and tracking throughout their care journey.

These exercises provided valuable insights into our capabilities and highlighted areas for improvement (Table 1).

**Table 1:** Table summarizing challenges and proposed remedies for different exercise types

	**Issues**	**Areas for improvement**
**Level 1**	Rapid mobilization of staff	Developing effective alert systems: an informal phone-based messaging group dedicated to crisis alert Crisis management team is responsible for individually and repeatedly contacting all teams
**Level 2**	Resource constraints and supply chain management	Establishing efficient protocols for blood product delivery Developing a “mobile pharmacy box” for rapid drug delivery
**Level 3**	Inefficient communication Organizational bottlenecks in patient care Role of leadership Identity traceability	Creating dedicated leadership roles to oversee patient flow and communication Conducting regular debriefing sessions to identify and address organizational issues Healthcare team dedicated to crisis management Dedicated personnel

## Fortius: together to success!

Within the anesthesiology and critical care departments, some physicians undergo simulation training. A working group has been established to oversee exercises and conduct debriefing sessions following simulations. A directive structured interview format was utilized for debriefing sessions, particularly during level 2 exercises with few participants. For each level 3 exercise, two debriefing sessions were conducted: an immediate post-exercise session to gather initial impressions and a follow-up session several days later to discuss areas for improvement. Experienced simulation facilitators were present during levels 2 and 3 exercises to identify and report areas for improvement. Experienced simulation facilitators conducted direct observations of participants, using checklists to assess both medical skills and organizational abilities related to patient flow within the hospital post-admission. Quantitative data collected during the exercises included the number of available personnel and their estimated arrival time at the hospital, the number of announced patients, the number of patients admitted to the resuscitation room, the number of operating rooms available, the number of ICU patients transferred to accommodate post-operative patients requiring resuscitation, and the final hospitalization location for each patient. The working group analyzed this data to identify any organizational bottlenecks in the hospital’s victim care pathway. Simulated victims were portrayed by actors and volunteer medical students. A psychiatrist from our institution was present during level 3 scenarios and actively participated in a post-exercise debriefing session with the actors, students who had assumed the role of victims, and participants. A significant limitation of our experience is that we have not conducted a formal evaluation of our disaster simulation system over the past 2 years. Our approach is informed by the recommendations of academic societies.

In conclusion, the simulation-based training scheme we have been running for the past 2 years improves inter-professional and inter-department communication both day-to-day and during crisis periods. Our experience highlights the importance of a well-structured team during mass casualty events. The key message is that some team members should focus on system-wide management rather than direct patient care. This allows for a more efficient allocation of resources and a better overall response to the crisis.

## Data Availability

No datasets were generated or analysed during the current study.
